# Olea Europœa

**Published:** 1854-11

**Authors:** 


Olea Europœa.—Mr. Maltas, in the Pharmaceutical Journal says, he has found *Olive leaves* more effectual in intermittent fever than the quinine. He tried it in the island of Mytilene, at a time when the disease was very prevalent. He boiled two handfuls of the leaves in a quart of water, until evaporation had reduced it to a pint; and then gave a wine-glassful every three or four hours.

